# Terminalia Catappa Extract (TCE) Reduces Proliferation of Lung and Breast Cancer Cell by Modulating miR-21 and miR-34a Expressions

**DOI:** 10.31557/APJCP.2021.22.4.1157

**Published:** 2021-04

**Authors:** Habib Zarredar, Amir Mahdi Khamaneh, Fatemeh Firouzi Amoodizaj, Dariush Shanehbandi, Ensiyeh Seyedrezazadeh, Hamed Sabagh Jadid, Milad Asadi, Venus Zafari, Yeganeh Khalili, Zahra Soleimani, Atefeh Ansarin, Majid Khalili

**Affiliations:** 1 *Tuberculosis and Lung Disease Research Center, Tabriz University of Medical Science, Daneshgah Street, Tabriz, Iran. *; 2 *Tuberculosis and Lung Disease Research Center & Rahat Breathe and Sleep Resaerch, Center, Tabriz University of Medical Science, Tabriz, Iran. *; 3 *Department of Genetics, Tabriz Branch, Islamic Azad University, Tabriz, Iran. *; 4 *Immunology Research Center, Tabriz University of Medical Sciences, Tabriz, Iran. *; 5 *Department of Basic Oncology, Institute of Health Sciences, Ege University, Izmir, Turkey. *

**Keywords:** Metastasis, Terminalia Catappa, breast cancer, apoptosis, lung cancer

## Abstract

**Background:**

After cardiovascular illness, cancer is the one of the main and second cause of death in the worldwide. Despite significant advances in this field, low survival, drug resistance, and side effects of chemotherapy remain an unsolved problem. Due to the high mortality rate among cancer patients, finding the new substance to treatment with low side effects is important. Previous studies have been informed that positive effects of herbal medicines on cancer patients, which are very efficient in the treatment of cancer.

**Methods:**

In this study, the antitumor effect of ethanolic *Terminalia catappa* leaf extract (TCE) on MCF-7, MDA-231, and A549 cell lines was examined. For this reason, the effects of TCE on cell migration, gene expression, and growth were investigated by scratch, test, real-time PCR (qPCR) qPCR, and MTT tests respectively.

**Results:**

As a reported by the MTT outcomes, TCE significantly decreased the viability of A549, MCF-7, and MDA-231 cells (P < 0.05). Moreover, genes expression patterns that are related to proliferation (miR-21, miR-34a), migration (MMP-13, Vimentin), and apoptosis (Cas-3, Cas-8, Cas-9, Bcl-2, Bax) also have changed significantly after treatment with TCE. Also, in the A549 cell line, Bax (p value: 0.029), Cas-9 (p value: 0.00023), miR-34a (p value: 0.031), Bcl-2 (p value: 0.0076), MMP-13 (p value: 0.041), Cas-3 (p value: 0.00051) and in MCF-7 cell line Bax (p value: 0.0004), Cas-3 (p value: 0.0003), Cas-9(p value: 0.037), miR-34a (p value: 0.005), Bcl-2(pvalue:0.0007), mir-21(p value:0.016), MMP-13(p value: 0.011) and in MDA-231 cell line Bax(p value<0.0001), Cas-3(p value: 0.003), Cas-9(p value: 0.0004). mir-34a (p value:0.0019), Bcl-2(p value:0.0023), MMP-13(p value: 0.032) have significantly changed compare to control group.

**Conclusion:**

The outcomes of this research determined that T. Catappa might be a potential source of antitumor compounds and could be a candidate for further research.

## Introduction

Cancer is defined as an immoderate and uncontrolled progression of cells. Cancer cells are able to spread around tissues and metastasize to distant organs utilizing lymphatic system or blood vessel (Fidler, 1989; Rusciano, 1992). Mutations and irregular patterns of gene expression lead to abnormal gene function which is the main reason for cancer progression (Li et al., 2000). Annually, 10 million people die from cancer in the world (Saika and Sobue, 2013). Even though there is a remarkable improvement in early diagnosis and treatment of tumor, but still rate of mortality is high (Bertucci et al., 2012; Bray et al., 2012). 

Accordingly, this study focuses on two main types of cancer, namely breast and lung cancers, which are the most prevalent malignancies in humans. Breast cancer is the most prevalent type of cancer in women, with about half of million deaths and 1.5 million new cases every year (Ferlay et al., 2015). Lung cancer is the main reason of cancer-related deaths with 1,761,007 deaths (18.4% of the total cancer-related deaths) and 2,093,876 newly diagnosed cases in 2018 (Ferlay et al., 2010; Stewart, 2015). Currently, treatment protocol of cancer including surgery, chemotherapy, radiography, and immunotherapy is the basis of cancer treatment. Although, chemotherapy is a more commonly used method, it has several problems including toxicity, drug resistance, and limited effectiveness (Tan et al., 2011). 

On the other hand, herbal drugs have been used for centuries to treat and prevent diseases. In this regard, the *Terminalia catappa* (Combretaceae family) plant that is a native plant of India, has been studied for its anti-tumor properties (Ezeokonkwo, 2004). The previous studies have shown that T.catappa leaf extract (TCE) has antioxidant (Kinoshita et al., 2007), anti-inflammatory, anti-metastatic ,and anti-tumor effects (Fan et al., 2004; Yang et al., 2010). Also, punicalagin as the main part of TCE, has been found to have useful properties against bleomycin-induced genotoxicity in ovary cells of the Chinese hamster (Chen et al., 2000) and chemopreventive effect on H-ras-transformed NIH3T3 cells (Chen and Li, 2006). Also, TCE has been indicated to have effective antimutagenicity and has been more cytotoxic to human hepatoma cells compared to normal liver cells (Ko et al., 2003). 

Therefore, in this study, the apoptotic, anti-cancer, and cytotoxic effects of TCE on A549, MCF7, and MDA231 cell lines are investigated. Also, expression of important genes correlated with metastasis, proliferation, and apoptosis is assessed by quantitative polymerase chain reaction (qPCR).

## Materials and Methods

*Preparation of TCE*

In the present study, plant leaves were collected from Rudan City (Hormozgan Province, Iran). Healthy green leaves without pests and diseases were selected for the study. Then, 30 g of leaves was dried and powdered at 40 °C for 12 h. Then, the powdered leaves were dissolved in 50% ethanol and were extracted by maceration technique 3 times. In the next step, the vacuum rotary evaporator was used to separate solvent from the extracted compound to obtain TCE. Finally, for using TCE, 5mg of the extract was solved in dimethyl sulfoxide (DMSO) (100 μl) solution and then, was added to complete culture medium (900 μl).

*Cancer Cell Cultures*

MCF7, MDA 231, and A549 cancer cell lines were obtained from the Pasteur Institute of Iran (Tehran, Iran). The prepared cancer cells were cultured on RPMI1640 medium supplemented with 1% antibiotics including penicillin (100 IU/ml) plus streptomycin (100 μg /ml) (SigmaAldrich, USA) and fetal bovine serum (FBS) 10% (Biochrom, Berlin), under the condition of 5% CO_2 _atmosphere, 95% of humidity, and temperature of 37°C. All the experiments were done when the cells reached logarithmic phase.

*Cytotoxicity Assay*

A number of 104 cancer cells were seeded in 96-well plates. The cells were treated for 24-72 h at 37°C with different concentrations of TCE (0.5, 1, 2, 4, 8, 12, 16, 25, and 32 μg). Rate of cell viability after treatment by TCE was determined using the MTT assay. After 24-72 h of incubation, 100 μl of MTT reagent (5 mg/ml) solution was added to each well. Then, the plates were incubated for 4 h at 37°C. After incubation, the MTT reagent and medium in each well were removed, and Sorenson buffer plus DMSO (200 μl) were added to the wells and they were placed on a shaker incubator for 30 min. Optical density (OD) of each well was measured by the enzyme-linked immunosorbent assay (ELISA) reader (Bio-Tek, USA) at 570 nm. Moreover, IC_50_ level was determined by GraphPad Prism 6.0 software (GraphPad Software, USA).

*Scratch Test (Wound Healing Assay)*

In this method, MCF7, MDA231, and A549 cell lines were cultured on 6-well plates. Then, a sterile yellow pipette tip was used to create a scratch about 0.5 mm wide across the monolayer cell. After scratching, the detached cells were removed by washing with phosphate-buffered saline (PBS) solution and image was taken from the scratch region. Then, plates were treated with different concentrations of TCE and were incubated for 72 h. Images of the wounded area were recorded using a light microscope at time intervals of 0, 24, 48, and 72 h. Finally, rate of cell migration in the wounded area was calculated by evaluating width of the scratch region.

*Extraction of Total RNA, cDNA Synthesis, and qRT-PCR*

The total RNA extraction was done from the treated and untreated cell lines 24 h after TCE treatment using an extraction kit according to the manufacturer’s instructions (Takara Biotechnology Inc.). Takara cDNA synthesis kit (Takara, cat.No:RR037Q) was used for cDNA synthesis according to the manufacturer’s protocol. So, 2 μg of total extracted RNA was added to MMLV reverse transcriptase plus oligo-dT and random hexamer primers according to the manufacturer’s protocol. The StepOne Real-time PCR System (Applied Biosystems, USA) and the SYBR Green gene expression Master mix (Takara, Korea) were applied to perform quantitative analysis. For this reason, 1 μM of primer mix, 1.5 μl of cDNA template, 7.5μl of nuclease-free water, and 10 μl of SYBR green Premix were added to each micro tube. The glyceraldehyde-3-phosphate dehydrogenase (GAPDH) gene was used as an endogenous control. The primers were designed by Oligo 7 software (Table 1). Relative expression of *miR-21, miR-34a, Caspase 9, Caspase 8, vimentin, Bcl2, Caspase 3, matrix metalloproteinase-13 (MMP-13), *and *Bax *genes was determined by the 2 (-ΔΔCt) method (Livak and Schmittgen, 2001).

*Statistical Analysis*

All the data were expressed as mean ± standard deviation (SD). Statistical significance of variances between more than two samples were determined by analysis of variance (ANOVA) followed by Bonferroni’s and Sidak’s multiple comparisons and Unpaired Two-Sample Student’s t-test (P-values ˂ 0.05 were significant). Statistical analysis was done via Graph Pad Prism 6 (Graph Pad Software, USA). 

## Results

*Toxicity Effect of TCE on A549, MCF7, and MDA231 Cell Lines*

**Figure 1:**
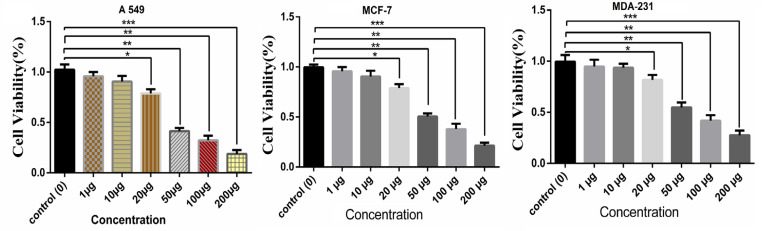
Effect of IC_50_ Concentration of TCE on A549, MCF-7, and MDA-231 Cell Proliferation: TCE decreased the cell survival to 17.6%, 21.4%, and 27.21 in 200 µg concentration compared to the control cells

For determining the cytotoxic effect of TCE on cancer cell viability, the A549, MCF7, and MDA231 cells were treated with different concentrations of TCE at time intervals of 24, 48, and 72h. Cell viability was considered by MTT assay in A549, MCF7, and MDA231cells, respectively. The results confirmed that the TCE decreased cell viability rate in a dose-dependent manner. Consequently, treating A549, MCF7, and MDA231 cell lines with 200 μg of the TCE reduced survival rate by 17.6, 21.4, and 27.21% , respectively compared to the control untreated cell (p < 0.05; Figure 1). Furthermore, an IC50 level was calculated by GraphPad Prism software 6.0 for A549 (42.32 μg), MCF7 (50.21 μg), and MDA-231 (53.87 μg) cell lines.

*Low Expression of MMP-13 and Bcl-2 and High Expression of miR-34a, Cas-9, Cas-3, and Bax in A549, MCF-7, and MDA-231 Cells after Treatment with TCE*

**Figure 2:**
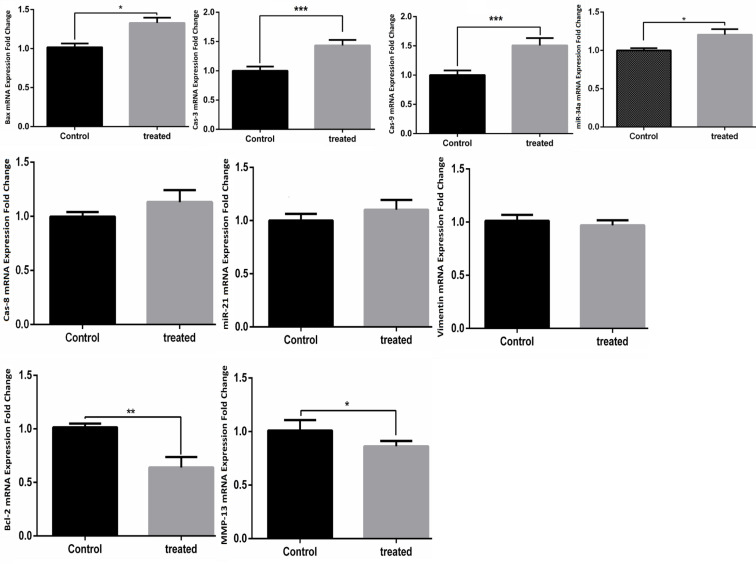
Effects of TCE Treatment on the Expression of BAX, Bcl-2, MMP-13, miR-21, miR-34a, Cas-3, Cas-8, Cas-9 and Vimentin in A549 Cell Line. The data was presented as mean ± SD (N=3) (* = P< 0.05).

**Figure 3:**
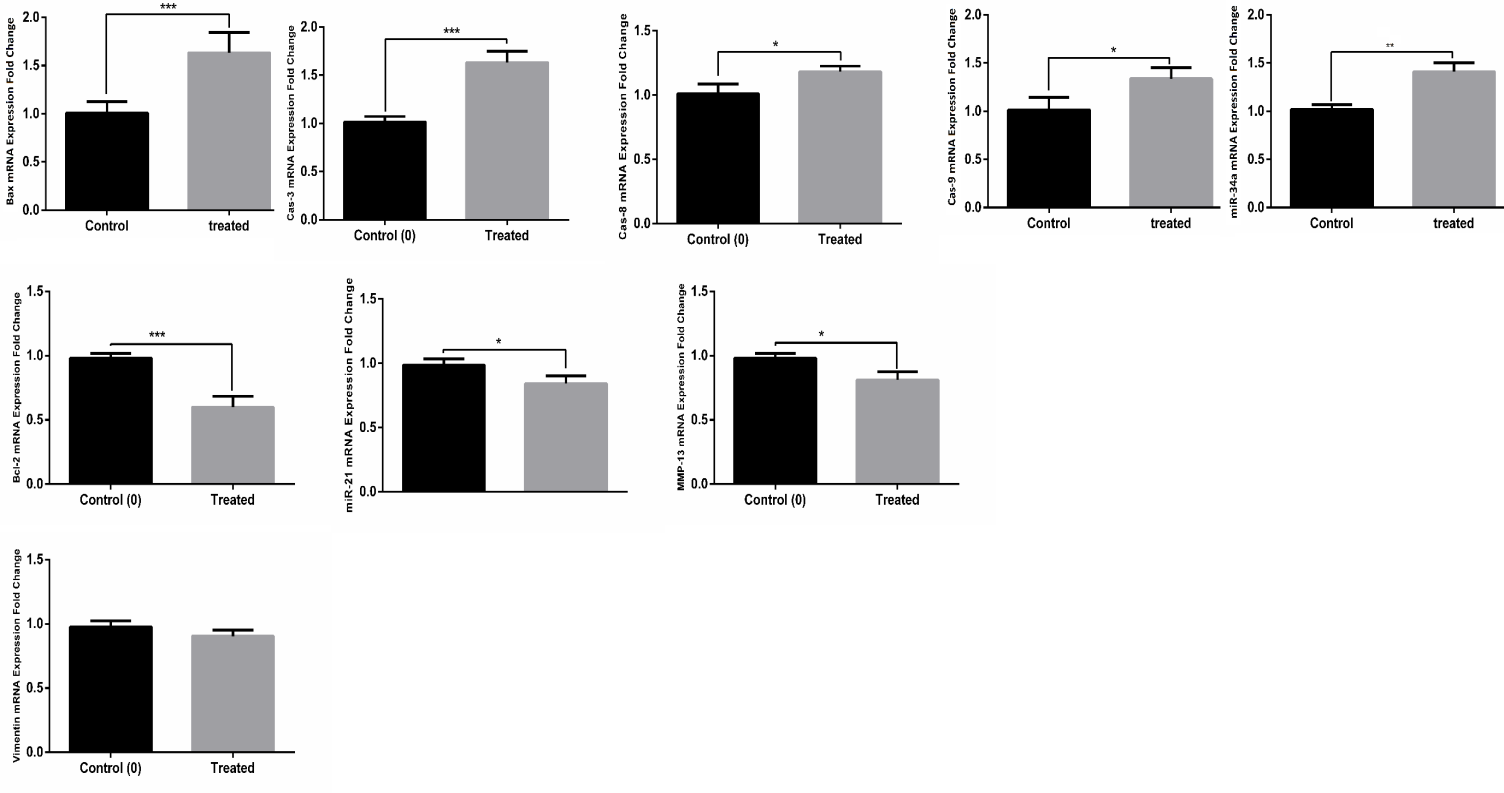
Effects of TCE Treatment on the Expression of BAX, Bcl-2, MMP-13, miR-21, miR-34a, Cas-3, Cas-8, Cas-9 and Vimentin in MCF-7 Cell Line. The data was presented as mean ± SD (N=3) (* = P< 0.05).

**Figure 4:**
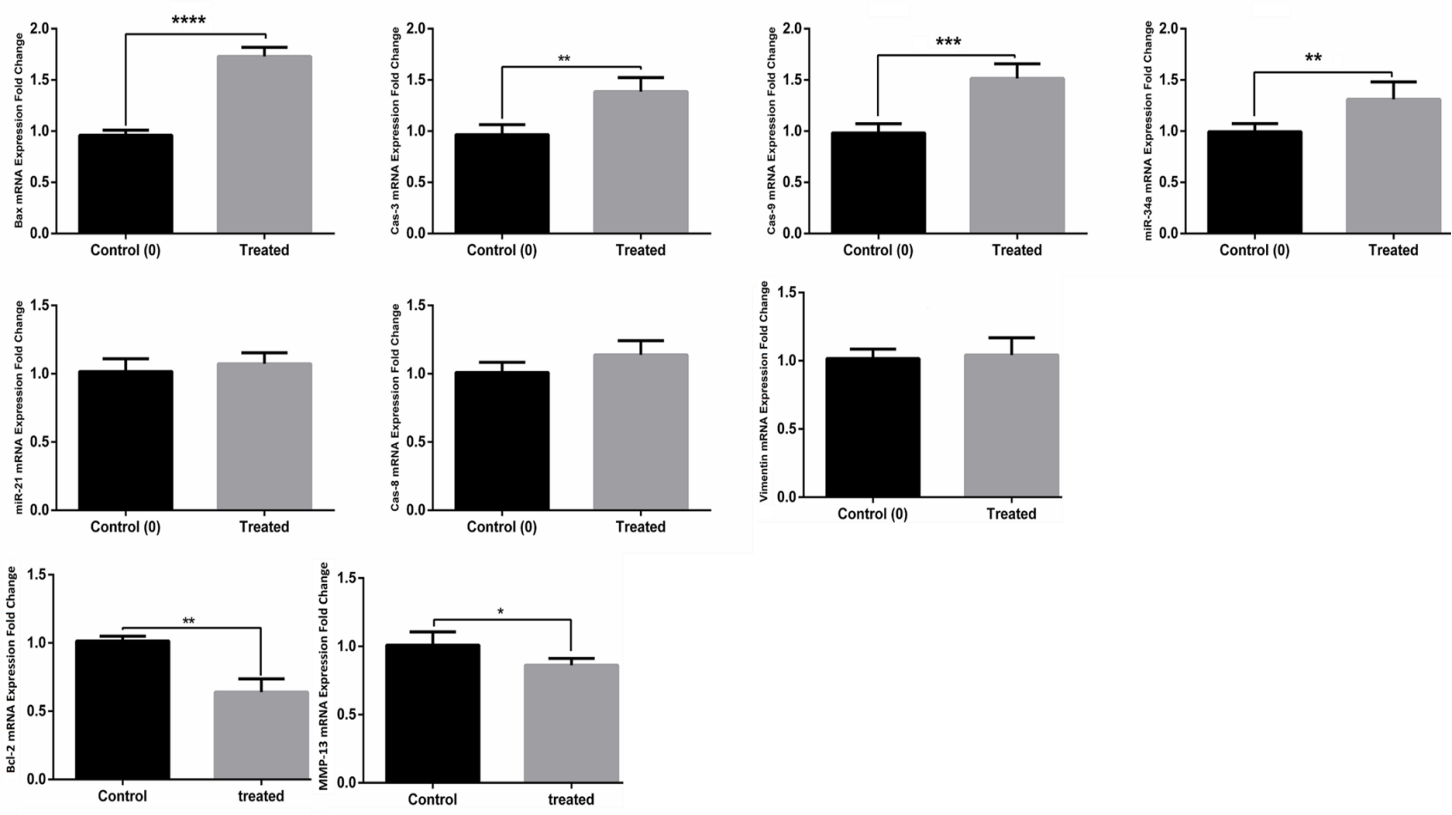
Effects of TCE Treatment on the Expression of BAX, Bcl-2, MMP-13, miR-21, miR-34a, Cas-3, Cas-8, Cas-9 and Vimentin in MDA-231 Cell Line. The data was presented as mean ± SD (N=3) (* = P< 0.05).

Effects of the TCE on expression level of *MMP-13, miR-21, Vimentin, Cas-8, Cas-3, miR-34a, Cas-9, Bax, *and *Bcl-2*, in A549 (Figure 2), MCF-7 (Figure 3), and MDA-231(Fig. 4) cell lines were determined by real-time PCR. In the MCF-7 cells, 24 h after treatment with* TCE, mRNA* expression level of the* miR-21* (p value: 0.016, fold change: 0.237), Bcl-2 (p value: 0.00078, fold change: 0.419) and MMP-13 (p value: 0.011, fold change: 0.273), was reduced by 76.3, 58.1 , and 72.7%, respectively. 

Also, mRNA level of miR-34a (p valu:0.0059, fold change:1.73), Cas-8 (p value: 0.017, fold change:1.21), Cas-3 (p value:0.00032 fold change:1.64), Cas-9 (p value:0.037, fold change:1.38), and Bax (p value:0.00043, fold change:1.61) was increased by 173, 121, 164, 138, and 161%, respectively. There was no significant change in the* mRNA* expression of Vimentin between control group and TCE-treated cells. 

In the A549 cells, 24 h after treatment with TCE, mRNA level of MMP-13 (p value:0.041, fold change:0.218) (p value: 0.041, fold change: 0.218) and Bcl-2 (p value:0.0076, fold change:0.381) (p value: 0.0076, fold change: 0.381) was reduced by 78.2 and 61.9%, respectively. Also, mRNA level of miR-34a (p value: 0.031, fold change:1.21), Bax (p value: 0.029, fold change:1.36), Cas-3 (p value: 0.00058, fold change:1.47), and Cas-9 (p value:0.00023, fold change:1.49) was increased by 121, 136, 147, and 149%, respectively. There was no significant change in* mRNA* expression level of *miR-21, Vimentin,* and *Cas-8* between control group and TCE-treated cells. 

In the MDA-231 cells, 24 h after treatment with TCE, mRNA expression level of *MMP-13* (p value:0.032, fold change:0.183) and Bcl-2 (p value:0.0023, fold change:0.439) was reduced by 81.7 and 56.1%, respectively. In the same way, expression level of *miR-34a* (p value:0.0019, fold change:1.31), Cas-3 (p value:0.003, fold change:1.41), Cas-9 (p value:0.00041, fold change:1.59), and Bax (p value<0.0001, fold change:1.78) was increased by 131,141, 159, and 178% ,respectively. There was no significant alteration in mRNA expression level of *Vimentin, miR-21*, and *Cas-8* between TCE-treated cells and control group.

*TCE Prohibited Migration of A549, MDA-231, and MCF-7Cell Lines*

**Figure 5:**
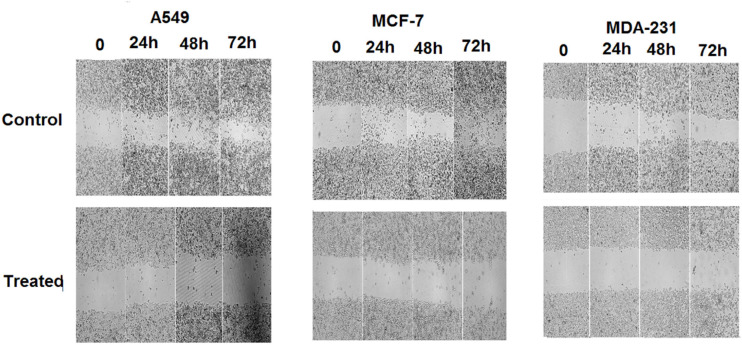
Results of Wound Healing Assay Shows that Untreated Cells (Control Group) have a Significant Further Migration in Comparison with Cells Treated with TCE

For determining the effect of the TCE on migration ability of the A549, MCF-7, and MDA-231 cells, after treating cell lines with the TCE, it was found that the treated cells did not have an ability to migrate and cover the scratch region. But the control group had the ability to migrate and cover the scratch area. Briefly, the results of wound healing assay indicated a notable decrease in capability of the TCE-treated cells in migration to the scratch region (Figure 5).

**Table 1:** Primer Sequences Used for Real-Time Gene Expression Quantification of the Target Genes and miRNAs

Primer	Forward and reverse	Sequence
Bax	Forward	5′- GACTCCCCCCGAGAGGTCTT-3′
	Reverse	5′- ACAGGGCCTTGAGCACCAGTT −3′
Bcl-2	Forward	5’- GAGCGTCAACCGGGAGATGTC -3′
	Reverse	5′- TGCCGGTTCAGGTACTCAGTC-3′
Caspase 3	Forward	5′-ATGGTTTGAGCCTGAGCAGA-3′
	Reverse	5′-GGCAGCATCATCCACACATAC-3′
β-actin	Forward	5’-TCCCTGGAGAAGAGCTACG-3′
	Reverse	5’-GTAGTTTCGTGGATGCCACA-3′
Caspase 9	Forward	5′-GCAGGCTCTGGATCTCGGC-3′
	Reverse	5′-GCTGCTTGCCTGTTAGTTCGC-3′
Caspase 8	Forward	5′-ACCTTGTGTCTGAGCTGGTCT-3′
	Reverse	5′--GCCCACTGGTATTCCTCAGGC -3′
miR-21	Exiqon	Product number:202,007
miR-34a	Exiqon	Product number: 202,860
MMP-13	Forward	5′-TGCAGAGCGCTACCTGAGATCATAC-3′
	Reverse	5′- GGAGCTTGCTGCATTCTCCTTCA-3′

## Discussion

For the years, herbal medicine has played important roles in preventing and treating various ailments, as well as cancer and chronic diseases. In this regard, importance of innovative technologies as well as gene editing (Ahmadzadeh et al., 2019) and siRNA technologies (Shanehbandi et al., 2019; Zarredar et al., 2019) has not been able to reduce importance of herbal medicine as a source of therapeutic agents. Anti-cancer herbal drugs have received great attention to find harmless therapeutics with low toxicity. Suppressive effect of the TCE has been described on invasion, migration, and proliferation of various tumor cells including lung cancer (Chu et al., 2007; Shanehbandi et al., 2019), hepatocellular carcinoma (Ko et al., 2003), and oral cancer (Yang et al., 2010). Thus, in the present study, it was hypothesized that treatment with the TCE may repress development, growth, and invasion ability of A549, MCF-7, and MDA-231 cell lines. Likewise, Yeh et al.,(2012) in their study confirmed the suppressive effect of the TCE on hepatocellular carcinoma (Yeh et al., 2012). Also, Lee et al., (2019) reported that the TCE had low cytotoxic effect on human HeLa and SiHa cervical cancer cells. Though, TCE has been found to inhibit mitogen-activated protein kinase (MAPK) and matrix metalloproteinase 9(MMP-9) pathway and decrease invasion and migration abilities of tumor cells (Lee et al., 2019). Also, Shanehbandi et al., (2020) found that the TCE promoted apoptosis, suppressed growth and migration rate in SW480 cell line by regulating expression of genes correlated with apoptosis and metastasis (Shanehbandi et al., 2019). In this research, the TCE displayed significant suppressive effects on proliferation, progression, and migration of A549, MCF-7, and MDA-231 cell lines. The final goal of the most therapeutic techniques for tumor treatment including radiotherapy and chemotherapy is promoting apoptosis and reducing cell development (Shekari et al., 2019). Apoptosis-related genes have essential role in numerous malignancies, particularly in cancer. In this study, after treating cancer cell lines by the* TCE, Cas-9 *expression was upregulated as the indicator of intrinsic apoptosis in all of the three cell lines Nevertheless, Cas-8 as a mediator of extrinsic apoptosis induction did not display a significant expression in the *A549* and MDA-231 treated cells. But, Cas-8 displayed significant upregulation in the MCF-7 cell line. Also, in this study, after treatment with the TCE, Cas-3 which is related to mitochondrial and extrinsic apoptosis signaling pathways showed a significant upregulation. According to results of *qRT-PCR*, expression level of the* Bcl-2* was reduced in the treated cancer cells. On the other hand, *Bax* expression level was upregulated in the treated cells. Bakhshaiesh et al., (2015) indicated that Bax/Bcl2 ratio might be a marker of susceptibility to apoptosis (Bakhshaiesh et al., 2015). Moreover, in this study MMP-13 as a vital member of the MMP family was meaningfully less expressed. Yang et al., (2010) demonstrated that the TCE could inhibit invasion and metastases of SCC-4 oral cancer by suppressing expression of urokinase plasminogen activator (u-PA), matrix metalloproteinase 2(MMP-2), and MMP-9 both in protein and mRNA levels (Yang et al., 2010). The remarkable role of MMP-13 in initiation of the extracellular MMP cascade has been proposed in the recent studies (Bakhshaiesh et al., 2015). Also, Chu et al., (2007) showed that the TCE had a dose-dependent suppressive effect on invasion ability and metastasis of Lewis lung carcinoma and A549 cells. Additionally, in the TCE-treated cell lines, expression level of *miR-34a *(as a tumor suppressor) was upregulated (A549, MCF-7, and MDA-231). The miR-34a had suppressive effect on proliferation and invasion of tumor cells by activation of p53. Bcl-2 mRNA was also targeted by MiRNA-34a as an important factor in cancer cells (Chu et al., 2007). So, after treatment with the *TCE*, expression levels of *miR-21 *and *Vimentin* (as a metastasis gene) were reduced. But, rate of change was not statistically significant. Therefore, according to the previous findings and results of the current study, the TCE might have ability to control malignancy and promote apoptosis in A549, MDA-231, and MCF-7cell lines.

In conclusion, our findings revealed that the TCE suppressed growth and induced apoptosis in A549, MCF-7, and MDA-231 cell lines. Also, the *TCE* reduced metastases and migration ability of cell lines by regulating expression level of genes correlated with metastasis and apoptosis. So, the TCE can be used as a candidate anti-cancer agent in treatment of lung and breast cancers.

*List of Abbreviations*

TC = *Terminalia Catappa*

TCE = *Terminalia Catappa* Extract

MMP = Matrix metalloproteinase

## Author Contribution Statement

Habib Zarredar: Conceptualization, Visualization, Supervision. Amir Mahdi Khamaneh: Methodology, Software, Validation. Fatemeh Firozi Amoodizal: Software, Validation. Milad Asadi: Methodology, Software, Editing. Venus Zafari: Software, Validation. Ensiyeh Seyedrezazadeh: Software, Validation. Yeganeh Khalili: Software, Writing. Zahra Soleimani: Visualization, Writing. Hamed Sabagh Jadid: Visualization, Writing, Editing. Atefeh Ansarin: Visualization, Writing. Majid Khalili: Conceptualization, Methodology, Validation. Dariush Shanehbandi: Methodology, Supervision.
